# Variation of life‐history traits of the Asian corn borer, *Ostrinia furnacalis* in relation to temperature and geographical latitude

**DOI:** 10.1002/ece3.2275

**Published:** 2016-06-26

**Authors:** Liang Xiao, Hai‐Min He, Li‐Li Huang, Ting Geng, Shu Fu, Fang‐Sen Xue

**Affiliations:** ^1^ Institute of EntomologyJiangxi Agricultural UniversityNanchangChina; ^2^ Jiangxi Entry Exit Inspection and Quarantine BureauNanchangChina; ^3^ Institute of Plant ProtectionChinese Academy of Agricultural SciencesBeijingChina

**Keywords:** Body weight, development time, geographical populations, growth rate, *Ostrinia furnacalis*, sexual size dimorphism, temperature

## Abstract

Life‐history traits from four geographical populations (tropical Ledong population [LD], subtropical Guangzhou [GZ] and Yongxiu populations, and temperate Langfang population [LF]) of the Asian corn borer, *Ostrinia furnacalis* were investigated at a wide range of temperatures (20–32°C). The larval and pupal times were significantly decreased with increasing rearing temperature, and growth rate was positively correlated with temperature. The relationship between body weight and rearing temperature in *O. furnacalis* did not follow the temperature–size rule (TSR); all populations exhibited the highest pupal and adult weights at high temperatures or intermediate temperatures. However, development time, growth rate, and body weight did not show a constant latitudinal gradient. Across all populations at each temperature, female were significantly bigger than males, showing a female‐biased sexual size dimorphism (SSD). Contrary to Rensch's rule, the SSD tended to increase with rising temperature. The subtropical GZ population exhibited the largest degree of dimorphism while the temperate LF exhibited the smallest. Male pupae lose significantly more weight at metamorphosis compared to females. The proportionate weight losses of different populations were significantly different. Adult longevity was significantly decreased with increasing temperature. Between sexes, all populations exhibit a rather female‐biased adult longevity. Finally, we discuss the adaptive significance of higher temperature‐inducing high body weight in the moth's life history and why the moth exhibits the reverse TSR.

## Introduction

Most insects are ectothermic (cold‐blooded animals). Their body temperature varies directly with environmental temperature. When food is not limiting, the heat is the force driving the rate of growth and development. Therefore, increasing temperature within a favorable range will speed up the metabolism of insect and consequently accelerate development rate. As a generally accepted guideline, increased temperature can lead to higher growth rates, shorter development times, and smaller adult size in insects and other ectotherms (Sibly and Atkinson 1994). This phenomenon has been called the “temperature–size rule (TSR)” and has been observed in more than 80% of ectothermic species studied, and has been found in various organisms including animals, plants, protozoa, and bacteria (Atkinson 1994). As with all biological “rules,” however, some exceptions to the TSR exist, including well‐documented cases of the reverse TSR, where the body sizes increased with temperature (Mousseau and Roff 1989; Atkinson 1995; Kingsolver et al. 2007). Interestingly, most of the known exceptions to the rule have been found in insects (including Lepidoptera) (Atkinson 1994). In 67 insect species, 18% showed significant increases in size with increased temperature, and 7% showed size was largest at intermediate temperature (Atkinson 1994). However, we are still unknown what force drives most insect species following the TSR, and the minority of insects exhibiting the reverse TSR.

Species distributed over broad geographic ranges often display thermal clines in body size, with the majority of species showing larger adult size in colder environments (Partridge and French 1996). This geographic variation in body size is consistent with the intraspecific version of Bergmann's rule, which declares that strains of a species tend to be larger in colder environments (Blackburn et al. 1999; Ashton 2004). Bergmann size clines accord with the reaction norms described by the TSR because temperature changes systematically with latitude, a gradient in temperature is supposed to create the increase in body size with increasing latitude (Partridge and Coyne 1997). Like the TSR, exceptions to Bergmann's rule exist. A number of insects exhibited that their body sizes decrease with increasing latitude or altitude (so‐called converse Bergmann cline) (Mousseau 1997; Blanckenhorn and Demont 2004; Dillon et al. 2006; Gaston and Chown 2010). Size declines with latitude in some insects are not constant, but adopt the form of a saw‐tooth cline, such that increasing season length results in increasing body size until two generations can be merged within a season, at which point the body size descends precipitously (Roff 1980; Masaki 1996; Nygren et al. 2008). No latitudinal size variation has been found in some lepidopterous insects (Gaston and Chown 2010; Barton et al. 2014).

Female and male animals are rarely the same size, a phenomenon called sexual size dimorphism (SSD). Females are usually larger than males in insects, and only 7% of insect species with SSD have larger males than females (Stillwell et al. 2010). Because the SSD is generally produced by different selection pressures between sexes (Masaki 1967; Landman et al. 1989; Mousseau and Roff 1989; Roff and Mousseau 2005; Berner and Blanckenhorn 2006; Bidau and Martía 2007), the SSD shows differences between temperatures and among populations inhabiting different environments (David et al. 1994; Blanckenhorn and Demont 2004; Blanckenhorn et al. 2006; Stillwell et al. 2007, 2010; Stillwell and Fox 2009). Understanding body size variation between the sexes can be helpful to predicted relationship between size and SSD. A common empirical relationship between body size and SSD among species is called Rensch's rule (Fairbairn 1997). Rensch's rule describes the observation that male‐biased SSD increases and female‐biased SSD decreases with body size (Rensch 1950; Abouheif and Fairbairn 1997; Fairbairn 1997). In most insect species, sexual size difference was shown to increase with increasing body size in species with female‐biased SSD (Teder and Tammaru 2005).

That longevity decreased with increasing temperature has been reported in many insects (Norry and Loeschcke 2002a,b; Dhileepan et al. 2005; Kuo et al. 2006; Norry et al. 2006; Karl and Fischer 2009). This frequent pattern is because metabolic rates increase under high temperature in ectotherms (Hulbert et al. 2004; Terblanche et al. 2005; Finkler 2006); consequently, higher metabolic rates generally result in shorter lifespan (Speakman 2005; Melvin et al. 2007). However, adult longevity in *Helicoverpa armigera* (Hübner) was reduced at mean fluctuating temperatures 17.5 and 32.5°C, but was longest at 25°C (Mironidis 2014). A sex‐specific variation for the mean longevity has also been found in *Drosophila melanogaster* Meigen with males living longer than females at 25°C and females living longer than males at 14°C (Norry and Loeschcke 2002a,b). Variation in longevity may also be related to geographic abiotic factors. That longevity in the wild varies with latitude, and altitude may also be linked to temperature. For example, the lifespan of the fly *D. melanogaster* from North America showed counter‐gradient variation with an increase in mean lifespan along a latitudinal range from 25 to 44°N (Schmidt and Paaby 2008). By contrast, the *D. melanogaster* populations from eastern Australia (collected among 25–44°S) showed linearly decreased longevity with increasing latitude (co‐gradient variation) (Sgrò et al. 2013). However, the longevity in Copper butterfly, *Lycaena tityrus* did not exhibit an altitudinal pattern (Karl and Fischer 2009). The evolutionary mechanisms underlying the clinal patterns in longevity are still unclear, but could be associated with an evolutionary adaptation to latitudinal or altitudinal changed environmental conditions.

The Asian corn borer, *Ostrinia furnacalis* (Guenée) (Lepidoptera: Crambidae), a serious economic pest to maize production, is widely distributed in maize‐producing regions throughout China. This borer enters facultative diapause as fully grown larvae in response to short‐day length during the autumn (Du and Cai 1964; Gong et al. 1984; Shen et al. 1988; Dai et al. 2000; Yang et al. 2014). This widely distributed species encounters great diversity in different localities and exhibits considerable diversity in life history among different geographic populations. Populations differ clearly in voltinism, the critical daylength for diapause induction, cold hardiness, and postdiapause emergence time (Lu et al. 1995; Ma et al. 2008; Xia et al. 2012; Huang et al. 2013; Fu et al. 2015; Xiao et al. 2015). However, little is known about the variations in development time, body size, growth rate in relation to temperature, and latitude in *O. furnacalis* (Tu et al. 2011). Development time, body size, and growth rate are three key life‐history traits. These traits should typically be of great importance for fitness because they reflect insect adaptation to environment (Nylin and Gotthard 1998).

In this study, we collected the Asian corn borer, *O. furnacalis* from four geographical regions which represent tropical, subtropical, and temperate zones of China to test the variation of life‐history traits at a wide range of temperatures. We obtained life‐history data of development time, body size, growth rate, and longevity from four geographical populations in relation to temperature and geographical latitude. Our results provide empirical evidences for the reverse TSR and Rensch's rule. These data may help us to understand evolutionary importance of life‐history variation.

## Materials and Methods

### Insect culture

Four populations of *O. furnacalis* used in this investigation were the Ledong population (LD) with seven generations per year from Ledong county (18°47′N, 108°89′E) (tropical LD population) Hainan Province (Wang et al. 2000), the Guangzhou population (GZ) with 5–6 generations per year from Guangzhou city (23°8′N, 113°17′E) (subtropical population), Guangdong Province (Li et al. 2014), the Yongxiu population (YX) with 3–5 generations per year from Yongxiu county (29°04′N, 115°82′E), Jiangxi Province (subtropical population) (Shen et al. 1988), and the Langfang population (LF) with 2–3 generations per year from Langfang (39°32′N, 116°40′E) (temperate population), Hebei province (Yu and Yu 2013). About 40 females in each population were collected from corn fields in August 2012 in the four regions. Adults were placed into plastic bags with 10% of honey water to produce egg masses. After hatching, larvae were transferred to plastic boxes (diameter 12 cm, height 15 cm) and reared on an artificial diet (Qiao et al. 2008) under a diapause‐averting photoperiod of LD 18:6 h at 25°C until larvae matured, then they were placed individually in cell culture plates with 24 holes (for each hole: diameter: 1.5 cm; height: 2 cm) for pupation and eclosion. The second of the laboratory‐reared generation was used in the experiments. All experiments were performed in illuminated incubators (LRH‐250‐GS; Guangdong Medical Appliances Plant, Guangdong, China). The light intensity during photophase was approximately 1.97 W·m^**−**2^, and the variation in temperature was ±1°C.

### Pre‐adult life‐history traits

The newly hatched larvae from all the four populations were reared at seven temperatures (20, 22, 24, 26, 28, 30, and 32°C) under LD 16:8. For all the individuals, we recorded the development time from hatching to pupation and adult eclosion. We calculated the pupal weight on the second day after pupation using an electric balance (AUY120 produced by SHIMADZU Corporation, Kyoto, Japan). Growth rate was calculated as ln pupal weight/larval development time. SSD for pupae was estimated for each population at each temperature using the Lovich and Gibbons (Lovich and Gibbons 1992) index, in which SSD = (size of the larger sex/size of the smaller sex) − 1, made positive when females are the larger sex and negative when males are the larger sex. Overall, 8984 offspring from the four populations were raised to adult. Sample sizes are between 133 and 577 for each temperature of each population.

### Adult life‐history traits

For all populations at each temperature, more than 100 newly eclosed adults that emerged in early period were placed individually in a small plastic box (diameter: 3.5 cm; height: 5 cm) after the release of the meconium and weighed with an electric balance. We calculated the proportionate weight loss between pupation and adult eclosion using the formula given in Gotthard et al. (1994): Proportion weight lost = l − (adult weight/pupal weight).

The SSD was estimated for each population at each temperature using the Lovich and Gibbons (Lovich and Gibbons 1992) index. After weighing, adults were still maintained in their own plastic box and their own temperature without providing any food to observe their lifespan. The total sample sizes are 3152. Sample sizes are between 99 and 135 for each temperature of each population.

### Statistical analyses

Life‐history traits were analyzed in relation to sex, temperature (20, 22, 24, 26, 28, 30, and 32°C) and population (LD, GZ, YX, and LF) with GLM. One‐way analysis of variance (ANOVA) was used to determine whether there were significant differences in life‐history traits in different populations at each temperature. One‐way ANOVA and Tukey's test were used to compare the differences in life‐history traits between sexes in each population and each temperature. Longevity was ln‐transformed before analyzing by survival analysis (Cox regression). The Cox proportional hazard model *h*_*i*_(*t*) = *h*_0_(*t*)*e* ^*ƞi*^ (model 1) (Collett 2003) was used to assess the effect of temperature, population, and sex on survival patterns. All statistical analyses were performed with R 3.02 (R Core Team, 2013).

## Results

### Pre‐adult life‐history traits

**Figure 1:**
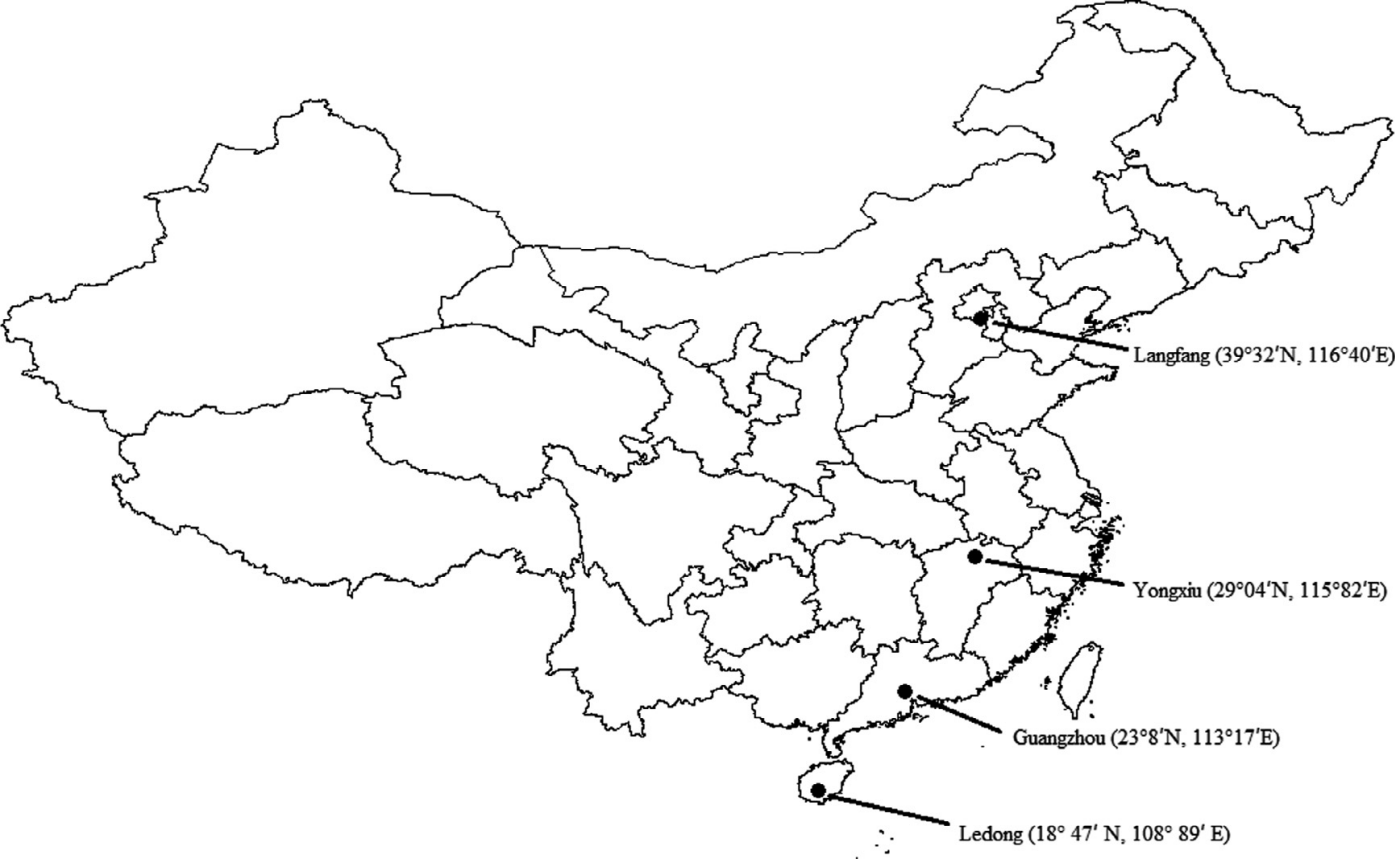
The location of collection sites of samples of *Ostrinia furnacalis*.

Almost all the life‐history traits were significantly influenced by temperature, population, sex, and their interactions (temperature × population × sex) (Table 1; Fig. 1).

**Table 1:** Results from a linear model analysis of fixed effects on larval time, pupal time, larval + pupal time, and growth rate in *Ostrinia furnacalis* in relation to sex, temperature, and population

Traits	Fixed effects	df	*F*	*P*
Larval time	Temperature	6	2929.571	<0.001
	Population	3	183.221	<0.001
	Sex	1	279.534	<0.001
	Population × temperature	18	29.308	<0.001
	Population × sex	3	19.254	<0.001
	Temperature × sex	6	9.350	<0.001
	Population × temperature × sex	18	2.865	<0.001
Pupal time	Temperature	6	14,512.926	<0.001
	Population	3	40.850	<0.001
	Sex	1	389.771	<0.001
	Population × temperature	18	36.819	<0.001
	Population × sex	3	9.966	<0.001
	Temperature × sex	6	4.976	<0.001
	Population × temperature × sex	18	1.545	0.065
Larval + pupal time	Temperature	6	4851.860	<0.001
	Population	3	176.000	<0.001
	Sex	1	167.300	<0.001
	Population × temperature	18	30.510	<0.001
	Population × sex	3	19.794	<0.001
	Temperature × sex	6	8.103	<0.001
	Population × temperature × sex	18	2.870	<0.001
Pupal weight	Temperature	6	67.416	<0.001
	Population	3	304.448	<0.001
	Sex	1	7404.966	<0.001
	Population × temperature	18	17.502	<0.001
	Population × sex	3	71.627	<0.001
	Temperature × sex	6	22.527	<0.001
	Population × temperature × sex	18	3.058	<0.001
Growth rate	Temperature	6	4019.862	<0.001
	Population	3	100.410	<0.001
	Sex	1	14.894	<0.001
	Population × temperature	18	23.590	<0.001
	Population × sex	3	10.637	<0.001
	Temperature × sex	6	6.146	<0.001
	Population × temperature × sex	18	2.525	<0.001

**Figure 2:**
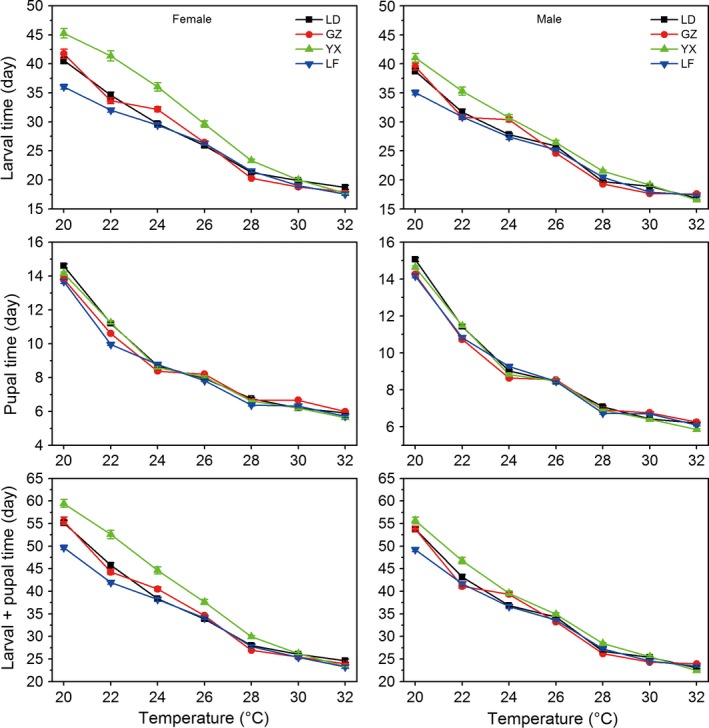
Larval development time, pupal time, and total development time (larval + pupal time) in *Ostrinia furnacalis* females and males in relation to temperatures and populations (LD: tropical Ledong population; GZ: subtropical Guangzhou population; YX: subtropical Yongxiu population; LF: temperate Langfang population). Error bars indicated SE.

Larval time, pupal time, and larval + pupal time of both females and males for all populations were significantly decreased with increasing rearing temperature (Fig. 2, Table 1 for temperature main effect). Female larvae required more days to develop than males at all temperatures, whereas male pupae required more days to develop than females at all temperatures (Fig. 2, Table 1 for sex main effect, see also Table S1). Except at 26°C for the tropical LD population and at 32°C for the temperate LF population, the total development times (from hatching to adults) in females were longer than in males at all temperatures (Fig. 2, Table 1 for sex main effect; see also Table S1). Total development time did not show a constant latitudinal gradient, the subtropical YX population had the longest developmental times at 20, 22, 24, 26, 28, and 30°C but not at 32°C (Fig. 2, Table 1 for temperature × population effect). Total development time was shortest in the temperate LF population at 20°C.

**Figure 3:**
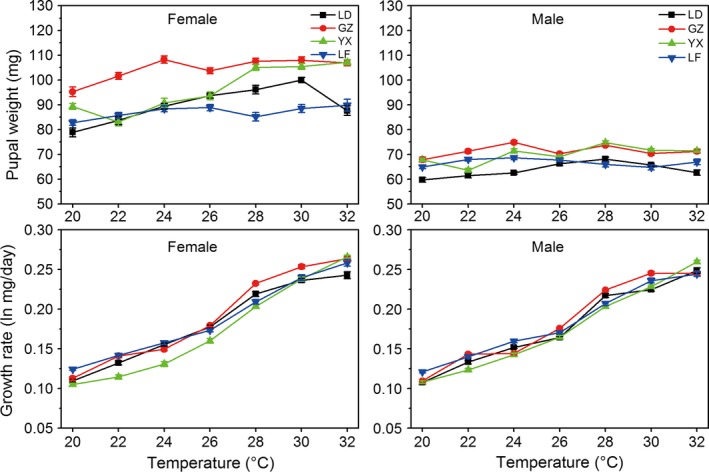
Pupal weight and larval growth rate in *Ostrinia furnacalis* females and males in relation to temperatures and populations (LD: tropical Ledong population; GZ: subtropical Guangzhou population; YX: subtropical Yongxiu population; LF: temperate Langfang population). Error bars indicated SE.

**Figure 4:**
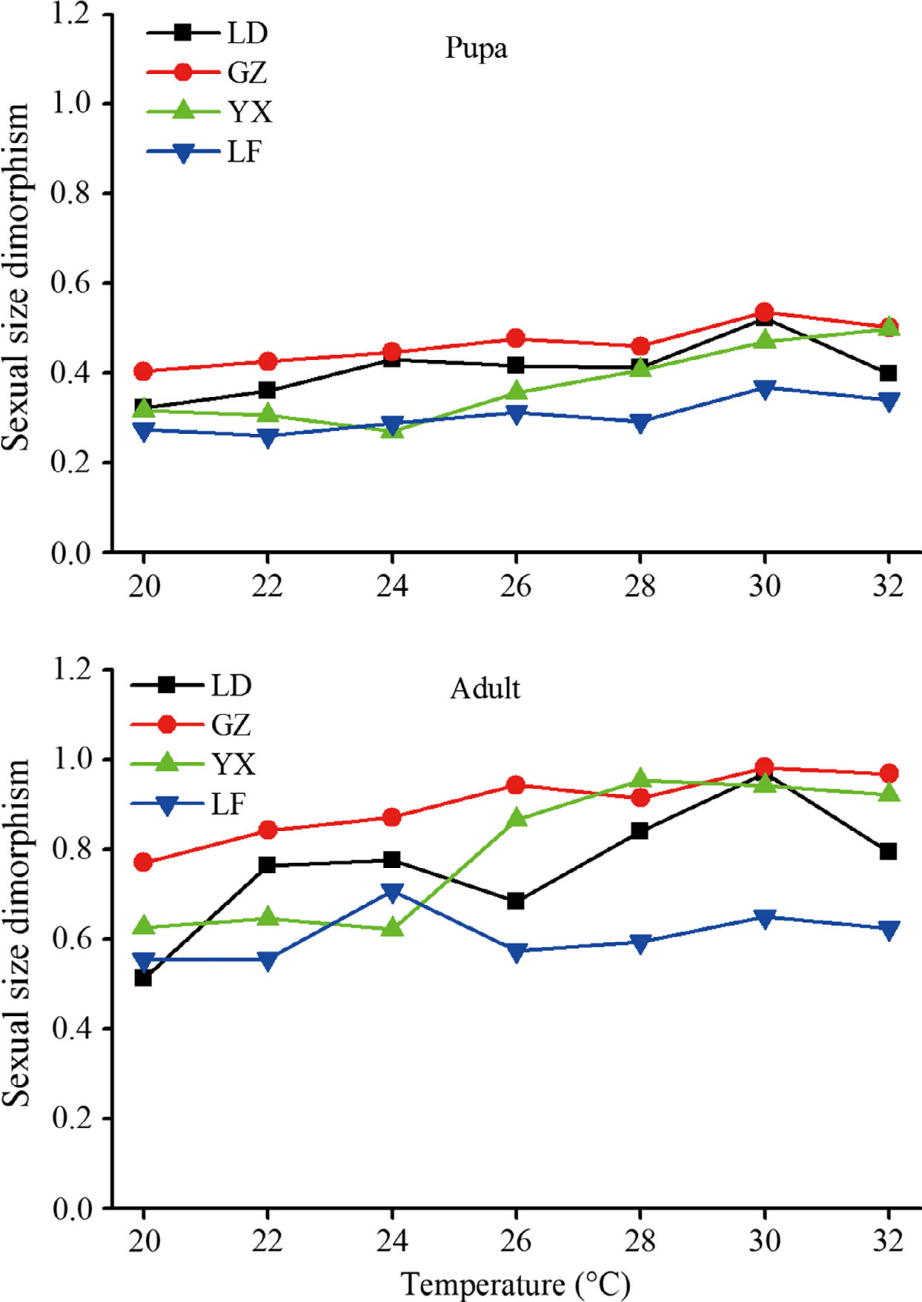
Sexual size dimorphism of populations of *Ostrinia furnacalis* reared at seven different temperatures (20, 22, 24, 26, 28, 30, and 32°C). Sexual dimorphism was estimated for each population at each temperature as (mean size of the larger sex [mg]/mean size of the smaller sex [mg]) − 1, made positive when females were the larger sex and negative when males were the larger sex. Error bars indicated SE.

Pupal weight was significantly influenced by temperature, population and sex, showing a result of significant temperature × population × sex interaction (Table 1). Female pupal weight was gradually increased with increasing rearing temperature from 20 to 30°C in the tropical LD population, form 22 to 32°C in the subtropical YX population, from 20 to 26°C in the temperate LF population, and from 20 to 24°C in the subtropical GZ population (Fig. 3; Table 1 for temperature main effect). Female pupal weight was highest at 24°C in the GZ population and at 32°C in the LF population (Table 1 for temperature × population effect). The influence of temperature on male pupal weight was some different from female pupal weight. Male pupal weight showed a gradually increase with increasing temperature from 20 to 28°C in the LD population, from 20 to 24°C in the GZ population (Fig. 3). Male pupal weight was highest at 28°C in the YX population and at 24°C in the LF population. There were significant differences in pupal weight among different populations (Table 1). The GZ population had the highest female pupal weight at 20, 22, 24, 26, 28, and 30°C, whereas the LF population had the lowest female pupal weight at 24, 26, 28, and 30°C (Fig. 3). Across all populations at each temperature, female pupae were significantly bigger than males (26–53.6% heavier than males, Fig. 3; see also Table S2), showing a female‐biased SSD. Sexual size dimorphism for all population tended to increase with rising temperature (Fig. 4); for example, SSD was 0.32, 0.40, 0.32, and 0.28 at 20°C in the LD, GZ, YX, and LF populations, respectively, whereas SSD was increased to 0.52, 0.54, 0.47, and 0.37 at 30°C, respectively. The subtropical GZ population exhibited the largest degree of dimorphism while the temperate LF exhibited the smallest (Fig. 4).

For the two sexes; growth rate in all populations was positively correlated with temperature (Fig. 3). Growth rate did not show a constant latitudinal gradient. The temperate LF population had the highest growth rate at 20 and 24°C, significantly higher than the other three populations at 20°C (female: *F*_3,536_ = 31.162, *P *<* *0.05; male: *F*_3,600_ = 18.416, *P *<* *0.05), whereas at 26, 28, and 30°C, the highest growth rate was found in the subtropical GZ population, significantly higher than the other three populations at 26°C (female: *F*_3,685_ = 15.131, *P *<* *0.05; male: *F*_3,754_ = 10.465, *P *<* *0.05). The significant differences of growth rate between the sexes were found at 22 and 24°C for subtropical YX population (at 22°C: *t *=* *2.734, df = 290.025, *P *<* *0.05; at 24°C: *t *=* *3.180, df = 190.785, *P *<* *0.05), at 30°C for tropical LD, GZ, and YX populations (LD: *t *=* *3.764, df = 388.664, *P *<* *0.05; GZ: *t *=* *2.464, df = 353, *P *<* *0.05; YX: *t *=* *2.753, df = 440, *P *<* *0.05), at 32°C for GZ, YX, and temperate LF populations (GZ: *t *=* *5.388, df = 344, *P *<* *0.05; YX: *t *=* *2.118, df = 368, *P *<* *0.05; LF: *t *=* *2.807, df = 165, *P *<* *0.05), in which females had significantly higher growth rate than males except YX population at 22, 24, and 26°C, in which the growth rate was significantly higher in males than females (Table S2).

### Adult life‐history traits

Like pupal weight, there were also significant impacts of temperature, population, and sex on adult weight (Table 2, Fig. 4).

**Table 2:** Results from a linear model analysis of fixed effects on adult weight, proportionate weight loss in *Ostrinia furnacalis* in relation to sex, temperature, and population

Traits	Fixed effects	df	*F*	*P*
Adult weight	Temperature	6	5.537	<0.001
	Population	3	184.516	<0.001
	Sex	1	7928.557	<0.001
	Population × temperature	18	7.230	<0.001
	Population × sex	3	11.865	<0.001
	Temperature × sex	6	66.783	<0.001
	Population × temperature × sex	18	3.540	<0.001
Proportionate weight loss	Temperature	6	19.219	<0.001
	Population	3	58.432	<0.001
	Sex	1	5095.211	<0.001
	Population × temperature	18	3.881	<0.001
	Population × sex	3	10.598	<0.001
	Temperature × sex	6	13.693	<0.001
	Population × temperature × sex	18	2.555	<0.001

Among temperatures, females achieved highest body weight at 28°C in the LD population, whereas male weight was increased with increasing temperature from 22 to 28°C. The highest adult weight was found at 24°C for both females and males in the GZ population. Female weight was increased with increasing temperature from 22 to 28°C in YX population, whereas males gained the highest body weight at 24°C. Female weight was increased with increasing temperature from 20 to 24°C in LF population, whereas male weight was highest at 22°C (Fig. 4, see also Table S3).

Among populations, the GZ population exhibited the highest female weight at 20–24°C, whereas the YX population had the highest adult weight at 26–32°C, significantly higher than LD and LF populations (26°C: *F*_3,210_ = 26.824, *P *<* *0.05; 28°C: *F*_3,248_ = 40.707, *P *<* *0.05; 30°C: *F*_3,217_ = 25.841, *P *<* *0.05; 32°C: *F*_3,202_ = 23.778, *P *<* *0.05). For male weight, the YX population exhibited the highest weight at 24–32°C (Fig. 4, see also Table S3). It should mention that adult weight change with temperature was not completely similar to pupal weight change; this may be due to samples (only the early period emerging adults were weighed).

**Figure 5:**
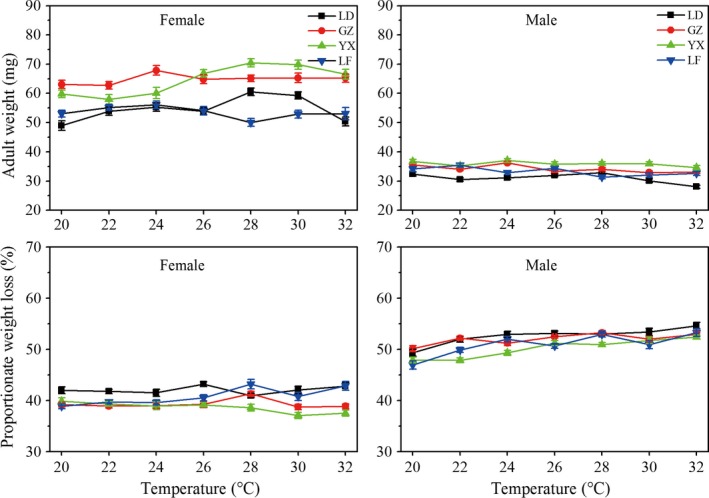
Adult weight and proportionate weight loss of *Ostrinia furnacalis* females and males in relation to temperatures and populations (LD: tropical Ledong population, GZ: subtropical Guangzhou population, YX: subtropical Yongxiu population, LF: temperate Langfang population). Error bars indicated SE.

Between sexes, female adults were significantly bigger than males (51.2–98.3% heavier than males, Fig. 5; see also Table S3). Furthermore, the differences of body weight between sexes increased with increasing temperature; for example, SSD was 0.51, 0.77, 0.63, and 0.55 at 20°C in the LD, GZ, YX, and LF populations, respectively, whereas SSD was increased to 0.97, 0.98, 0.94, and 0.65 at 30°C, respectively. Figure 5 also shows that male pupae lose significantly more weight at metamorphosis compared to females (see also Table S3). Thus, this temperature‐dependent size dimorphism was more pronounced in the adult stage than at the beginning of the pupal phase (Fig. 4).

**Figure 6:**
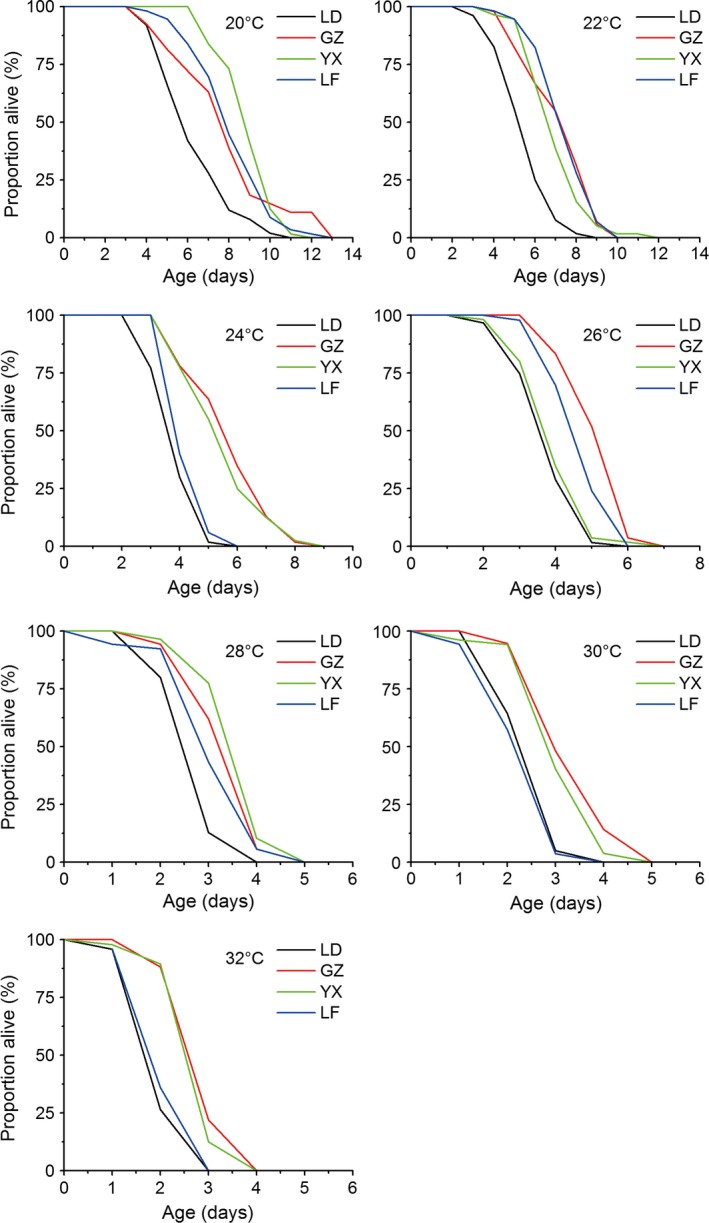
Survival curves of *Ostrinia furnacalis* females from four populations at each experimental temperature.

**Figure 7:**
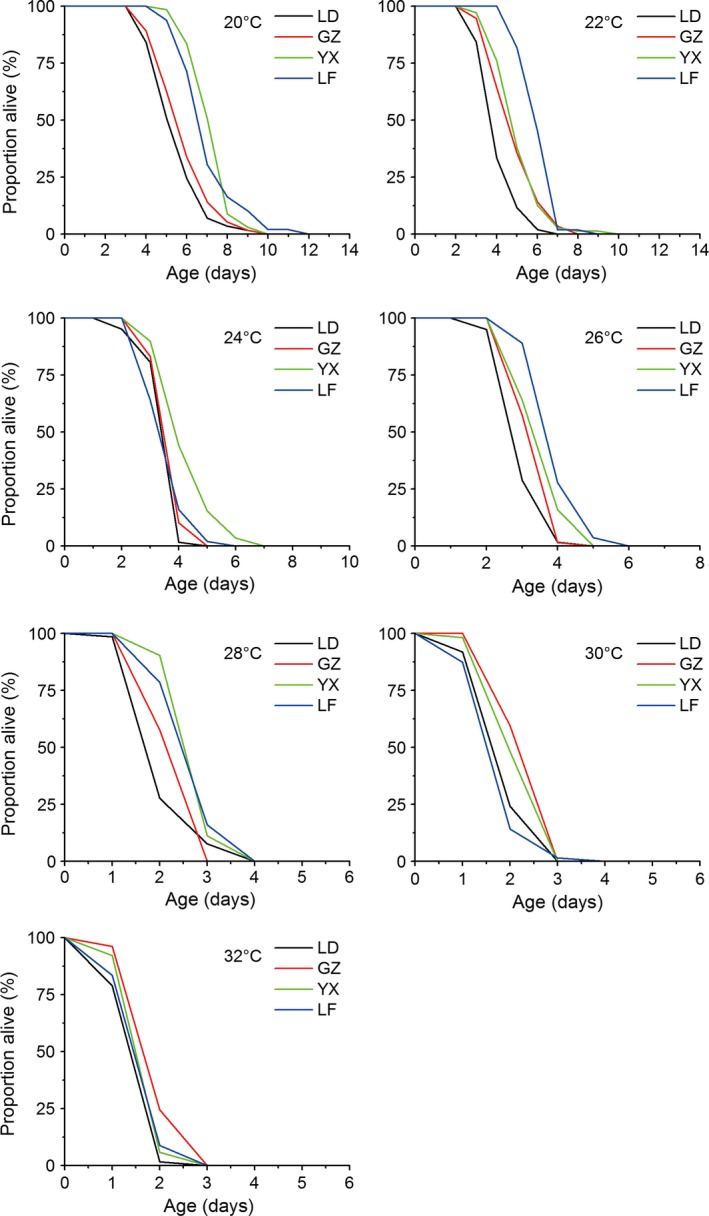
Survival curves of *Ostrinia furnacalis* males from four populations at each experimental temperature.

Adult longevity was also significantly influenced by temperature, population, sex, and their and their interactions (temperature × population and population × sex) (Table 3). Adult longevity for both female and male in all populations was significantly decreased with increasing temperature (Table 3 for temperature main effect). Between sexes, all populations exhibit a rather female‐biased adult longevity at all temperatures, average longevity was significantly longer in female than male (Table S3). Adult longevity was significant different among populations at each temperature. The significant temperature × population interaction indicates that the differences in longevity among populations varied when temperature changed; for example, the both females and males of the tropical LD population lived shortest at all temperatures except at 30°C with slightly longer longevity compared to the temperate LF population (Figs. 6, 7). The GZ population lived longest at 24, 26, 30, and 32°C for female adults and at 30 and 32°C for male adults. The YX population lived longest at 20 and 28°C for female adults and at 20, 24, and 28°C for male adults. The temperate LF population lived longest at 22°C for female adults and at 22 and 26°C for male adults (Figs. 6, 7). Interestingly, male longevity increased with increasing latitude at 22°C (from 4.3 to 6.3 days) and 26°C (from 3.2 to 4.2 days), showing a latitudinal gradient variation.

**Table 3:** Results of Cox proportional hazard analysis for survivorship in relation to population, temperature, and sex

Fixed effect	df	*χ* ^2^	*P*
Population	3	11.557	<0.01
Temperature	6	895.61	<0.001
Sex	1	9.353	<0.001
Population × temperature	18	84.364	<0.001
Temperature × sex	6	1.796	0.937
Population × sex	3	12.121	<0.01
Population × temperature × sex	16.410	0.0159	0.564

## Discussion

Probably the most intriguing result of our study is that the relationship between body weight and rearing temperature in *O. furnacalis* did not follow the TSR; all populations exhibited the highest body weights at high temperatures or intermediate temperatures (Figs. 3, 5). For example, Female pupal weight was gradually increased with increasing rearing temperature from 20 to 30°C in the tropical LD population, from 22 to 32°C in the subtropical YX population, from 20 to 26°C in the temperate LF population, from 20 to 24°C in the subtropical GZ population (Fig. 3). Male pupal weight showed a gradually increase with increasing temperature from 20 to 28°C in the LD population, from 20 to 24°C in the GZ population, and male pupal weight was highest at 28°C in the YX population and at 24°C in the LF population (Fig. 3). Body weight of adults also exhibited a similar temperature response tendency (Fig. 5). So far, most studies of the TSR used only 2–3 rearing temperatures, and so have limited power to decide the true shape of the thermal reaction norm (Kingsolver and Huey 2008). Our experiment used seven rearing temperatures (from 20 to 32°C) and showed that shorter development times caused by higher temperatures led to an increase of body weight, providing a good example of the reverse TSR. High body weight at higher temperature in *O. furnacalis* may reflect that biomass accumulation rate during larval development period is more temperature sensitive than development rate (Angilletta and Dunham 2003; Zuo et al. 2012); that is, increasing temperature has a stronger effect on growth rate than on development rate (Van der Have and Jong 1996). That higher temperature produced high body weight in *O. furnacalis* may be an evolutionary adaptation for subtropical and temperate populations to reach a higher larval weight at maturity before hibernation. According to our laboratory and field observations, the critical factor for diapause induction in *O. furnacalis* is photoperiod, with a much weaker effect of temperature (Du and Cai 1964; Gong et al. 1984; Yang et al. 2014; Fu et al. 2015; Xiao et al. 2015). In nature, such a response pattern allows individuals who hatched under high‐temperature conditions during late August for temperate population and early September for subtropical to enter diapause. Thus, most overwintering larvae developed at higher temperatures (not lower than 25°C) and gained higher body weight before hibernation. Undoubtedly, bigger larvae will tend to have higher survival rate during winter and greater performance after hibernation (Peters 1983; Chown 1995). Moreover, selection for higher body weight with increasing temperature at adult stage in nondiapause generation may enhance reproductive success, as adults with larger body weight will tend to have greater fecundity and fitness than smaller individuals within a population (Peters 1983; Stearns 1992; Roff 1992). Therefore, this result implies that global climate warming during autumn in temperate region will benefit the reproduction of this moth, and the pest will have the capacity to become greater threats. Furthermore, we think whether an insect species follows the TSR or not may be relation to its diapause character. That the Asian corn borer exhibits the reverse TSR is because its diapause induction is determined by photoperiod, which leads to the induction of larval diapause occurring in warmer autumn conditions. So the Asian corn borer experiences strong selection for body weight under warm conditions during evolution. The recent study with the rice stem borer, *Chilo suppressalis* (Walker), a species with a strong diapause response triggered by photoperiod and entering facultative diapause as fully grown larvae in response to short‐day conditions and high temperature during the autumn (Xiao et al. 2010), also reveals that the moth from a temperate region (44.93°N, 127.17°E) exhibits the reverse TSR with the highest body weight at 31°C in a range of temperature from 22 to 31°C. This temperate population is mainly univoltine. The first‐generation larvae occur in summer (from late June to early August) and are exposed to warm summer during diapause induction (Fu D.M., He H.M., Zou C., Xiao H.J., Xue F.S. for unpublished data). In the cabbage white butterfly, *Pieris rapae* (L.), body size increases with increasing rearing temperature in a population from North Carolina, because the North Carolina population experiences strong selection for body size under warm conditions (Kingsolver et al. 2007). In order to confirm our idea, we will investigate more insect species with similar diapause character.

Our experimental results indicate that the geographical variations of life‐history traits of the four populations in development time, body weight, and growth rate do not show a constant latitudinal gradient. Degree of geographical variation in these traits depends on temperature. For example, the subtropical YX female population had the longest total developmental times at temperatures from 20 to 30°C, significantly longer than the tropical LD population, subtropical GZ population, and the temperate LF population, whereas the temperate LF female population had the shortest total developmental times at 20–24°C (Fig. 2). The subtropical GZ population had the greatest female pupal weight at 20, 22, 24, 26, 28, and 30°C, whereas the temperate LF population had the smallest female pupal weight at 24, 26, 28, and 30°C (Fig. 3). The temperate LF population had the highest growth rate at 20 and 24°C, whereas the highest growth rate was found at 26, 28, and 30°C in the subtropical GZ population. The absence of a latitudinal gradient variation in these traits is surprising because the four experimental populations are distributed in distinctively geographical latitudes with a 20° latitudinal range. Interestingly, if we only use three populations (remove the tropical LD population or temperate LF population) to analyze life‐history data, we detect a latitudinal gradient in these traits. For example, larval development time for females increased with increasing latitude at 20, 24, and 26°C if removing the temperate LF population (Fig. 2). Pupal weight decreased with increasing latitude at 20, 24, 26, 28, and 30°C if removing the tropical LD population, following the Bergmann size clines. Growth rate was negatively correlated with latitude at 24°C but positively correlated with latitude at 32°C if removing the temperate LF population.

Rensch (1950) observed that SSD increases when male body size is larger than female body size and decreases when female body size is larger than male body size (Abouheif and Fairbairn 1997; Fairbairn 1997). This means that male body size varies or diverges more over evolutionary time than female body size. Contrary to Rensch's rule, the present study in *O. furnacalis* with female‐biased SSD showed that SSD for all populations tended to increase with rising temperature. Because body size generally increased with increasing temperature, sexual differences in size were shown to increase with increasing body size. Furthermore, male pupae lost significantly more weight at metamorphosis compared to females; this temperature‐dependent size dimorphism was more pronounced in the adult stage than at the beginning of the pupal phase (Fig. 4). Our results may suggest that female body weight varies or diverges more over evolutionary time than male body weight. Our results are consistent with the study results reported by Teder and Tammaru (2005), where SSD within species increases with body size in insects with female‐biased SSD. We also found that the degree to which dimorphism changed with temperature varied among populations; for example, the subtropical GZ population exhibited the largest degree of dimorphism in body weight while the temperate LF exhibited the smallest at almost all rearing temperatures (Fig. 4). Our results also reveal that the magnitude of dimorphism within populations can vary considerably with environmental conditions due to a sex difference in plasticity of body weight (Fischer and Fiedler 2000; Teder and Tammaru 2005; Stillwell et al. 2007, 2010; Stillwell and Fox 2009).

For all populations in *O. furnacalis*, females were significantly bigger than males; for example, females were 51.2–77.1% heavier than males at 20°C and 65.0–98.3% heavier than males at 30°C (see Table S3). Females were also lived significantly longer than males in all populations at each temperature; for example, females lived 0.8–1.8 days longer than males at 20°C and 0.5–1 days longer than males at 30°C (see Table S3). It seems that environmental conditions not only influence body weight but also influence adult longevity, creating an environmentally based positive correlation between these traits that has no underlying genetic basis (Norry and Loeschcke 2002a,b; Fox et al. 2003a,b). Similar result was also reported in the seed beetle, *Callosobruchus maculates* Fabricius (Fox et al. 2003a,b). The gender differences in lifespan may be due to differences in body weight between males and females (Samaras et al. 2002). In most insect species, females are larger than males and live longer than males. This is because that larger species generally have lower metabolic rates (Oklejewicz and Daan 2002; Rollo 2002). In the seed beetle, *Stator limbatus* (Horn 1873), virgin males lost mass faster than virgin females (Savalli and Fox 1998), suggesting that males either keep a higher metabolic rate than females or are less effective at averting water loss. Adult lifespan in *O. furnacalis* was significantly different among populations at each temperature. The degree of variation depended on temperature. The longevity in females was longest at 24, 26, 30, and 32°C for GZ population, at 20 and 28°C for YX populations, and at 22°C for LF population (Table S3), without showing a constant latitudinal gradient. Like above body weight variation, if removing the tropical LD population, female adult longevity was negatively correlated with latitude at 24, 26, 30, and 32°C; if removing the temperate LF population, adult longevity was positively correlated with latitude at 20°C. Male adult longevity showed a latitudinal gradient at 22°C with positively correlated with latitude (Table S3). If moving the temperate LF population, male adult longevity was positively correlated with latitude at 20, 22, 24, 26, and 28°C.

## Conclusions

That the relationship between body weight and rearing temperature in *O. furnacalis* exhibited the highest body weights at high temperatures or intermediate temperatures strongly suggests that natural selection for body size is to serve as a strategy to ensure overwintering larvae obtaining higher body weight before hibernation. Thus, an insect species that shows a photoperiod‐inducing diapause at high temperature may exhibit the reverse TSR. We speculate that global climate warming during autumn in temperate region will make the Asian corn borer to have the capacity to become greater threats. Variation of life‐history traits in this study did not show a constant latitudinal gradient. However, if we only use three populations (remove the tropical LD population or temperate LF population) to analyze life‐history data, we did detect a latitudinal gradient in larval time, pupal weight, growth rate, and longevity. To best understand the geographical variation of life‐history traits, our results emphasize the importance of performing a life‐history experiment with a wide range of populations under a range of environmental conditions.

## Conflict of Interest

None declared.

## Supporting information


**Table S1.** Life‐history data (mean ± 1 SE) for female and male of LD, GZ, YX and LF populations of *Ostrinia furnacalis* at different temperatures.
**Table S2.** Life‐history data (mean ± 1 SE) for female and male of LD, GZ, YX and LF populations of *Ostrinia furnacalis* at different temperatures.
**Table S3.** Life‐history data (mean ± 1 SE) for female and male of LD, GZ, YX and LF populations of *Ostrinia furnacalis* at different temperatures.
