# Supplementary material for: Variation of life‐history traits of the Asian corn borer, Ostrinia furnacalis in relation to temperature and geographical latitude
Source: Ecol Evol. 2016 Jun 26;6(15):5129–43. doi: 10.1002/ece3.2275 (PMC4984492; doi:10.1002/ece3.2275)
Supplement: Supplementary file 1 — Table S1. Life‐history data (mean ± 1 SE) for female and male of LD, GZ, YX and LF populations of Ostrinia furnacalis at different temperatures. Table S2. Life‐history data (mean ± 1 SE) for female and male of LD, GZ, YX and LF populations of Ostrinia furnacalis at different temperatures. Table S3. Life‐history data (mean ± 1 SE) for female and male of LD, GZ, YX and LF populations of Ostrinia furnacalis at different temperatures. [file ECE3-6-5129-s001.doc]

Table S1 Life-history data (mean ± 1 SE) for female and male of LD, GZ, YX and LF populations of *Ostrinia furnacalis* at different temperatures. Figures followed by the same letter do not differ significantly between sexes in the same population at the each temperature (One-way analysis of variance (ANOVA) and Tukey’s test, threshold for significance *P* < 0.05)

| T (°C) Trait | LD | |  | GZ | |  | YX | |  | LF | |
| --- | --- | --- | --- | --- | --- | --- | --- | --- | --- | --- | --- |
|  | Female | Male |  | Female | Male |  | Female | Male |  | Female | Male |
| 20 | N = 100 | N = 121 |  | N = 97 | N = 122 |  | N = 204 | N = 207 |  | N = 138 | N = 154 |
| Larval time (d) | 40.57 ± 0.57a | 38.73 ± 0.48b |  | 41.74 ± 0.82a | 39.52 ± 0.61b |  | 45.28 ± 0.82a | 41.05 ± 0.73b |  | 36.04 ± 0.40a | 35.07 ± 0.40a |
| Pupal time (d) | 14.59±0.12b | 15.06±0.10a |  | 13.82±0.12b | 14.26±0.10a |  | 14.16±0.08b | 14.64±0.07a |  | 13.67±0.12b | 14.17±0.10a |
| Larval+pupal time(d) | 55.16±0.60a | 53.79±0.50a |  | 55.57±0.86a | 53.79±0.65a |  | 59.44±0.85a | 55.69±0.75b |  | 49.71±0.48a | 49.24±0.46a |
| 22 | N=120 | N=106 |  | N=139 | N=138 |  | N=152 | N=165 |  | N=192 | N=168 |
| Larval time (d) | 34.56±0.56a | 31.69±0.50b |  | 33.65±0.49a | 30.39±0.40b |  | 41.36±0.90a | 35.29±0.68b |  | 32.03±0.36a | 30.86±0.41b |
| Pupal time (d) | 11.19±0.08a | 11.43±0.13a |  | 10.60±0.08a | 10.72±0.09a |  | 11.24±0.09a | 11.47±0.10a |  | 9.95±0.06b | 10.83±0.08a |
| Larval+pupal time(d) | 45.75±0.59a | 43.12±0.55b |  | 44.25±0.53a | 41.12±0.45b |  | 52.60±0.94a | 46.76±0.72b |  | 41.98±0.39a | 41.69±0.45a |
| 24 | N=259 | N=318 |  | N=160 | N=167 |  | N=106 | N=121 |  | N=211 | N=163 |
| Larval time (d) | 29.66±0.31a | 27.81±0.24b |  | 32.15±0.42a | 30.80±0.40b |  | 36.03±0.73a | 30.75±0.48b |  | 29.44±0.40a | 27.39±0.44b |
| Pupal time (d) | 8.69±0.05b | 9.03±0.04a |  | 8.38±0.06b | 8.55±0.06a |  | 8.59±0.08b | 8.82±0.08a |  | 8.77±0.06b | 9.26±0.06a |
| Larval+pupal time(d) | 38.35±0.32a | 36.84±0.25b |  | 40.53±0.43a | 39.35±0.40b |  | 44.62±0.75a | 39.57±0.49b |  | 38.21±0.42a | 36.65±0.45b |
| 26 | N=167 | N=168 |  | N=189 | N=219 |  | N=143 | N=159 |  | N=190 | N=212 |
| Larval time (d) | 25.92±0.26a | 25.84±0.23a |  | 26.43±0.30a | 24.61±0.23b |  | 29.61±0.55a | 26.43±0.38b |  | 26.28±0.27a | 25.20±0.26b |
| Pupal time (d) | 7.95±0.05b | 8.44±0.05a |  | 8.20±0.05b | 8.63±0.05a |  | 8.03±0.07b | 8.47±0.06a |  | 7.81±0.05b | 8.46±0.05a |
| Larval+pupal time(d) | 33.87±0.26a | 34.28±0.23a |  | 34.63±0.31a | 33.24±0.23b |  | 37.64±0.56a | 34.91±0.37b |  | 34.09±0.28a | 33.67±0.27a |
| 28 | N=126 | N=121 |  | N=174 | N=192 |  | N=231 | N=234 |  | N=108 | N=115 |
| Larval time (d) | 21.29±0.30a | 19.69±0.22b |  | 20.27±0.15a | 19.29±0.11b |  | 23.34±0.24a | 21.53±0.19b |  | 21.49±0.26a | 20.50±0.24b |
| Pupal time (d) | 6.75±0.05b | 7.08±0.06a |  | 6.66±0.04b | 6.92±0.04a |  | 6.6±0.04b | 6.90±0.04a |  | 6.31±0.06b | 6.73±0.06a |
| Larval+pupal time(d) | 28.04±0.31a | 26.77±0.24b |  | 26.93±0.16a | 26.20±0.11b |  | 29.97±0.24a | 28.43±0.19b |  | 27.81±0.27a | 27.23±0.26a |
| 30 | N=202 | N=225 |  | N=166 | N=189 |  | N=197 | N=245 |  | N=76 | N=92 |
| Larval time (d) | 19.86±0.21a | 18.92±0.17b |  | 18.79±0.22a | 17.57±0.17b |  | 19.99±0.21a | 19.11±0.19b |  | 19.00±0.29a | 17.8±0.22b |
| Pupal time (d) | 6.20±0.04b | 6.43±0.04a |  | 6.66±0.05a | 6.76±0.05a |  | 6.19±0.05b | 6.41±0.04a |  | 6.37±0.06b | 6.70±0.05a |
| Larval+pupal time(d) | 26.06±0.21a | 25.35±0.18b |  | 25.45±0.24a | 24.33±0.18b |  | 26.18±0.23a | 25.51±0.20b |  | 25.37±0.32a | 24.58±0.23a |
| 32 | N=60 | N=73 |  | N=175 | N=171 |  | N=180 | N=190 |  | N=65 | N=102 |
| Larval time (d) | 18.72±0.34a | 16.86±0.26b |  | 17.93±0.16a | 17.67±0.17a |  | 17.73±0.13a | 16.57±0.12b |  | 17.57±0.26a | 17.36±0.19a |
| Pupal time (d) | 5.92±0.06b | 6.21±0.06a |  | 5.99±0.03b | 6.26±0.05a |  | 5.63±0.04b | 5.85±0.04a |  | 5.72±0.08b | 6.10±0.06a |
| Larval+pupal time(d) | 24.63±0.35a | 23.07**±**0.27b |  | 23.92±0.16a | 23.92±0.17a |  | 23.36±0.13a | 22.43±0.12b |  | 23.29±0.29a | 23.46±0.19a |

Table S2 Life-history data (mean ± 1 SE) for female and male of LD, GZ, YX and LF populations of *Ostrinia furnacalis* at different temperatures. Figures followed by the same letter do not differ significantly between sexes in the same population at the each temperature (One-way analysis of variance (ANOVA) and Tukey’s test, threshold for significance *P* < 0.05)

| T (°C) Trait | LD | |  | GZ | |  | YX | |  | LF | |
| --- | --- | --- | --- | --- | --- | --- | --- | --- | --- | --- | --- |
|  | Female | Male |  | Female | Male |  | Female | Male |  | Female | Male |
| 20 | N = 100 | N = 121 |  | N = 97 | N = 122 |  | N = 204 | N = 207 |  | N = 138 | N = 154 |
| Pupal weight (mg) | 78.85 ± 1.81a | 59.66 ± 0.91b |  | 95.24 ± 1.93a | 67.84 ± 0.71b |  | 89.33 ± 1.17a | 67.84 ± 0.71b |  | 82.82 ± 1.16a | 64.9 7± 73b |
| Growth rate(In mg/d) | 0.109±0.002a | 0.107±0.002a |  | 0.105±0.002a | 0.108±0.002a |  | 0.105±0.002a | 0.108±0.002a |  | 0.124±0.001a | 0.121±0.001a |
| 22 | N=120 | N=106 |  | N=139 | N=138 |  | N=152 | N=165 |  | N=192 | N=168 |
| Pupal weight (mg) | 83.65±1.65a | 61.46±0.92b |  | 101.60±1.37a | 71.24±0.78b |  | 82.98±1.58a | 63.55±0.78b |  | 85.69±1.16a | 67.99±0.75b |
| Growth rate(In mg/d) | 0.132±0.002a | 0.133±0.002a |  | 0.14a±0.002a | 0.143±0.002a |  | 0.114±0.003b | 0.123±0.002a |  | 0.141±.002a | 0.140±0.002a |
| 24 | N = 259 | N = 318 |  | N = 160 | N = 167 |  | N = 106 | N = 121 |  | N = 211 | N = 163 |
| Pupal weight (mg) | 89.39±1.15a | 62.51±0.55b |  | 108.27±1.44a | 74.84±0.67b |  | 90.72±1.88a | 71.39±0.89b |  | 88.43±1.22a | 68.61±0.81b |
| Growth rate(In mg/d) | 0.155±0.002a | 0.152±0.001a |  | 0.149±0.002a | 0.144±0.002a |  | 0.131±0.003b | 0.142±0.002a |  | 0.158±0.002a | 0.160±0.002a |
| 26 | N = 167 | N = 168 |  | N = 189 | N = 219 |  | N = 143 | N = 159 |  | N = 190 | N = 212 |
| Pupal weight (mg) | 93.76±1.17a | 66.17±0.74b |  | 103.71±1.15a | 70.23±0.59b |  | 93.60±1.58a | 68.98±0.74b |  | 88.84±1.31a | 67.72±0.67b |
| Growth rate(In mg/d) | 0.177±0.002a | 0.164±0.002b |  | 0.179±0.002a | 0.176±0.002a |  | 0.160±0.003a | 0.164±0.002a |  | 0.173±0.002a | 0.170±0.002a |
| 28 | N = 126 | N = 121 |  | N = 174 | N = 192 |  | N = 231 | N = 234 |  | N = 108 | N = 115 |
| Pupal weight (mg) | 96.07±1.72a | 68.04±0.81b |  | 107.51±1.25a | 73.66±0.56b |  | 105.09±1.22a | 74.75±0.65b |  | 85.15±1.71a | 65.93±1.00b |
| Growth rate(In mg/d) | 0.219±0.003a | 0.217±0.002a |  | 0.232±0.002a | 0.224±0.001b |  | 0.203±0.002a | 0.203±0.002a |  | 0.209±0.003a | 0.207±0.003a |
| 30 | N = 202 | N = 225 |  | N = 166 | N = 189 |  | N = 197 | N = 245 |  | N = 76 | N = 92 |
| Pupal weight (mg) | 100.00±1.07a | 65.71±0.58b |  | 108.03±1.21a | 70.31±0.64b |  | 105.46±1.20a | 71.67±0.68b |  | 88.53±1.59a | 64.72±1.00b |
| Growth rate(In mg/d) | 0.236±0.002a | 0.224±0.002b |  | 0.253±0.003a | 0.245±0.002b |  | 0.238±0.003a | 0.228±0.002b |  | 0.240±0.004a | 0.236±0.003a |
| 32 | N = 60 | N = 73 |  | N = 175 | N = 171 |  | N = 180 | N = 190 |  | N = 65 | N = 102 |
| Pupal weight (mg) | 87.61±1.98a | 62.64±1.13b |  | 106.87±1.17a | 71.17±0.62b |  | 107.15±1.12a | 71.49±0.71b |  | 89.82±2.42a | 66.96±1.14b |
| Growth rate(In mg/d) | 0.243±0.004a | 0.249±0.004a |  | 0.264±0.002a | 0.245±0.002b |  | 0.266±0.002a | 0.260±0.002b |  | 0.258±0.004a | 0.244±0.003b |

Table S3 Life-history data (mean ± 1 SE) for female and male of LD, GZ, YX and LF populations of *Ostrinia furnacalis* at different temperatures. Figures followed by the same letter do not differ significantly between sexes in the same population at the each temperature (One-way analysis of variance (ANOVA) and Tukey’s test, threshold for significance *p* < 0.05).

| T(°C) Trait | LD | |  | GZ | |  | YX | |  | LF | |
| --- | --- | --- | --- | --- | --- | --- | --- | --- | --- | --- | --- |
|  | Female | Male |  | Female | Male |  | Female | Male |  | Female | Male |
| 20 | N = 50 | N = 57 |  | N = 54 | N = 56 |  | N = 56 | N = 67 |  | N = 56 | N = 49 |
| Adult weight (mg) | 48.96 ± 1.63a | 32.38 ± 0.69b |  | 63.04 ± 1.40a | 35.60± 0.61b |  | 59.8 1 ± 1.29a | 36.76± 0.62b |  | 53.03 ± 1.14a | 34.11 ± 0.85b |
| Proporation weight loss | 41.99±0.69b | 49.31±0.67a |  | 39.18±0.49b | 50.12±0.61a |  | 39.88±0.63b | 47.88±0.55a |  | 38.86±0.46b | 46.92±0.77a |
| Adult longevity | 6.50±0.24a | 5.72±0.17b |  | 7.93±0.31a | 6.07±0.18b |  | 9.13±0.17a | 7.45±0.12b |  | 8.32±0.24a | 7.27±0.21b |
| 22 | N = 52 | N = 51 |  | N = 51 | N = 56 |  | N = 57 | N = 71 |  | N = 57 | N = 55 |
| Adult weight (mg) | 53.88±1.43a | 30.53±0.58b |  | 62.73±1.32a | 34.03±0.51b |  | 57.94±1.63a | 35.18±0.60b |  | 55.20±1.01a | 35.45±0.70b |
| Proporation weight loss | 41.75±0.44b | 51.96±0.55a |  | 38.91±0.42b | 52.15±0.40a |  | 39.32±0.46b | 47.86±0.45a |  | 39.70±0.39b | 49.86±0.50a |
| Adult longevity | 5.69±0.18a | 4.31±0.13b |  | 7.39±0.23a | 5.13±0.16b |  | 7.21±0.19a | 5.30±0.14b |  | 7.65±0.18a | 6.31±0.11b |
| 24 | N = 57 | N = 62 |  | N = 55 | N = 59 |  | N = 40 | N = 59 |  | N = 50 | N = 50 |
| Adult weight (mg) | 55.31±1.43a | 31.13±0.60b |  | 67.83±1.62a | 36.24±0.45b |  | 60.06±1.92a | 37.0 3±0.64b |  | 56.09±1.25a | 32.82±0.56b |
| Proporation weight loss | 41.50±0.69b | 52.92±0.59a |  | 39.00±0.56b | 51.20±0.46a |  | 38.94±0.77b | 49.29±0.50a |  | 39.61±0.50b | 51.98±0.56a |
| Adult longevity | 4.09±0.10a | 3.77±0.07b |  | 5.91±0.18a | 3.93±0.07b |  | 5.73±0.21a | 4.53±0.12b |  | 4.46±0.09a | 3.82±0.11b |
| 26 | N = 59 | N = 59 |  | N = 54 | N = 68 |  | N = 55 | N = 50 |  | N = 46 | N = 54 |
| Adult weight (mg) | 53.87±1.17a | 31.98±0.55b |  | 64.81±1.48a | 33.34±0.50b |  | 66.81±1.23a | 35.81±0.79b |  | 54.05±1.48a | 34.33±0.52b |
| Proporation weight loss | 43.17±0.45b | 53.13±0.48a |  | 39.23±.062b | 52.47±0.28a |  | 39.14±0.41b | 51.19±0.49a |  | 40.54±0.59b | 50.64±0.48a |
| Adult longevity | 4.02±0.11a | 3.25±0.07b |  | 5.39±0.11a | 3.59±0.06b |  | 5.16±0.13a | 3.80±0.10b |  | 4.91±0.12a | 4.20±0.09b |
| 28 | N = 70 | N = 65 |  | N = 71 | N = 59 |  | N = 58 | N = 62 |  | N = 53 | N = 56 |
| Adult weight (mg) | 60.48±1.30a | 32.84±0.54b |  | 65.15±1.08a | 34.02±0.55b |  | 70.38±1.40a | 36.01±0.56b |  | 50.06±1.33a | 31.40±0.68b |
| Proporation weight loss | 40.94±0.46b | 53.01±0.64a |  | 41.28±0.49b | 53.23±0.41a |  | 38.59±0.66b | 50.90±0.40a |  | 43.18±0.91b | 52.94±0.55a |
| Adult longevity | 2.93±0.07a | 2.34±0.08b |  | 3.62±0.08a | 2.58±0.06b |  | 3.84±0.08a | 3.02±0.06b |  | 3.36±0.12a | 2.95±0.08b |
| 30 | N = 59 | N = 62 |  | N = 56 | N = 52 |  | N = 52 | N = 54 |  | N = 54 | N = 63 |
| Adult weight (mg) | 59.27±1.19a | 30.09±0.64b |  | 65.29±1.61a | 32.92±0.57b |  | 69.81±1.54a | 35.94±0.61b |  | 52.90±1.33a | 32.06±0.76b |
| Proporation weight loss | 42.06±0.73b | 53.38±0.75a |  | 38.72±0.53b | 51.96±0.54a |  | 37.04±0.58b | 51.74±0.62a |  | 40.81±0.79b | 50.91±0.80a |
| Adult longevity | 2.69±0.07a | 2.16±0.07b |  | 3.57±0.11a | 2.60±0.07b |  | 3.35±0.11a | 2.46±0.07b |  | 2.56±0.09a | 2.03±0.07b |
| 32 | N = 49 | N = 57 |  | N = 59 | N = 53 |  | N = 48 | N = 51 |  | N = 50 | N = 67 |
| Adult weight (mg) | 50.33±1.51a | 28.04±0.54n |  | 65.20±1.43a | 33.11±0.51b |  | 66.54±1.68a | 34.60±0.76b |  | 53.03±2.11a | 32.64±0.74b |
| Proporation weight loss | 42.77±0.78b | 54.62±0.61a |  | 38.85±0.50b | 52.95±0.44a |  | 37.51±0.62b | 52.38±0.39a |  | 42.87±0.86b | 53.26±0.77a |
| Adult longevity | 2.22±0.07a | 1.81±0.06b |  | 3.10±0.08a | 2.21±0.07b |  | 3.00±0.08a | 1.98±0.05b |  | 2.32±0.08a | 1.93±0.06b |
